# Developing an obesity research agenda with British Pakistani women living in deprived areas with involvement from multisectoral stakeholders: Research priority setting with a seldom heard group

**DOI:** 10.1111/hex.13504

**Published:** 2022-04-28

**Authors:** Halima Iqbal, Jane West, Rosemary R. C. McEachan, Melanie Haith‐Cooper

**Affiliations:** ^1^ Born in Bradford Bradford Institute for Health Research, Bradford Teaching Hospital NHS Foundation Trust Bradford UK; ^2^ Faculty of Health Studies University of Bradford Bradford UK

**Keywords:** Bradford, coproduction, deprived areas, obesity research agenda, Pakistani women, research priority setting, seldom‐heard

## Abstract

**Introduction:**

British Pakistani women have exceptionally high rates of obesity and yet are seldom heard in a research priority setting concerning weight management. The objectives of this study were (i) to ascertain what multisectoral professionals perceive to be the most pressing unmet obesity needs or topic areas that need more research in relation to Pakistani women living in deprived areas of Bradford and (ii) to determine the top 10 obesity health priorities for this group to develop an obesity research agenda.

**Methods:**

A two‐step process was adopted using the following: (i) a survey of a wide range of multisectoral professional stakeholders (*n* = 159) and (ii) a ranking exercise involving Pakistani women living in deprived areas of Bradford (*n* = 32) to select and prioritize their top 10 obesity health concerns and unmet needs from a list of 31 statements identified in the survey and previous research. Survey data were analysed using inductive content analysis and themes were identified. Themes were translated into statements to be ranked by Pakistani women. The ranking exercise was conducted by telephone either via voice or video call. Data were analysed using a reverse scoring system.

**Results:**

Survey responses were grouped into statements reflecting the following three categories: education needs; healthy behaviour barriers and mental well‐being. The highest rankings were given by Pakistani women to statements on mental health and the need for education. The top 10 prioritized statements were developed with members of the public into an obesity research agenda that reflected the target population.

**Conclusion:**

Actively engaging British Pakistani women in setting research priorities provided a unique opportunity to understand the key areas they think are important for future research. The culminating research agenda can be used by researchers to advance the field of obesity research in Pakistani communities, thus producing research outputs that are relevant to and have impact in this population.

**Patient or Public Contribution:**

Participants in the ranking exercise collected data. Public contributors were involved in developing the prioritized statements into a research agenda.

## 1 INTRODUCTION

### 1.1 Obesity: Defining the problem

Obesity is a global public health issue. Carrying excess body fat increases an individual's susceptibility to diseases including cardiovascular disease, type 2 diabetes, respiratory conditions, muscular disorders and a range of psychological issues.^1^ More recently, obesity has been identified as a key risk factor for hospitalization and death due to COVID‐19 outcomes.^2^, ^3^, ^4^ British South Asian and other economically deprived populations have exceptionally high rates of obesity compared to the wider population.^5^ For instance, the prevalance of obesity is the highest in the most deprived 10% of the population—approximately double that of the least deprived 10%.^6^ The prevalence of obesity‐related disease, for example, type 2 diabetes, is reported to be up to six times higher in South Asian populations than in the wider population.^7^, ^8^, ^9^ Despite this, South Asian communities are less likely to follow a healthy diet or engage in exercise compared to the general population.^10^ Among the British South Asian population, the rates of obesity are higher in some subgroups, such as British Pakistani women (PW).^11^, ^12^ Bradford has a strong British Pakistani presence^13^ and is an economically deprived city in the north of England, which has higher obesity and Type 2 diabetes rates than the UK average.^14^, ^15^ These rates are pronounced in more deprived areas of the district where there is a large British Pakistani community.^16^

It is unclear whether efforts to reduce obesity in South Asian populations have been successful. For instance, one intervention positively changed the physical activity behaviours and attitudes of individuals from low socioeconomic groups living in deprived inner‐city areas of the United Kingdom^17^; yet, the ethnic profile of these individuals was not made explicit by the authors. Obesity reduction interventions specifically targeting British South Asian communities are scarce and the evidence from these interventions is mixed.^18^, ^19^, ^20^ It is more generally argued that for weight loss interventions for South Asian populations to be effective, they need to be culturally sensitive.^21^ There is an urgent need to address obesity in British PW. Development of a research agenda that has incorporated the perspectives of this population based on their lived experience could be one way to reduce the exceptionally high rates of obesity by developing obesity interventions and projects that more accurately reflect their obesity health needs.

### 1.2 Coproducing research agendas with ethnic minority populations

Research agendas have historically been determined by health service providers and funders.^22^ Debates continue on how to include a diverse range of voices in research design and delivery as part of the patient and public involvement.^23^ Marginalized groups are often uninvolved in research prioritization exercises and when they are involved, sample sizes are small and exercises are poorly conducted.^24^ Consequently, research questions that matter to people may be unheard,^25^ leading to failed interventions and alienation of already marginalized communities. This is especially concerning, given that these populations have disproportionately poorer health outcomes and can encounter negative experiences of services.^26^ Partnerships between professionals and the public to collectively identify and prioritize research by facilitated debate and formal decision‐making methods have been developed, such as the James Lind Alliance.^27^ However, holding consensus groups with underrepresented Black and minority ethnic individuals and professionals together may advantage professionals, leading to the disengagement of disadvantaged groups that are underrepresented in research priority setting.^28^, ^29^

Although research prioritization exercises in obesity do exist,^30^ no research could be found identifying the obesity research priorities of PW or South Asian women more generally. A research priority exercise involving South Asian children, young people and their families sought to prioritize health topics requiring more research according to this population.^31^ Nutrition, obesity and physical activity ranked highly as priority topics requiring further research. The objective of this study, therefore, was to produce an obesity research agenda that is reflective of PW's unmet needs, with added input from a range of professional stakeholders.

## 2 METHODS

### 2.1 Study design

**Figure 1:**
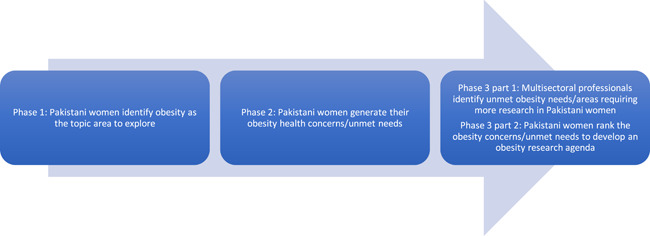
Phases of the research

Feminist participatory action research (FPAR) guided this study. FPAR provides an opportunity for women to voice their experiences of health and define health issues pertinent to them.^32^ This study is part of a wider research project influenced by earlier stages of the project (see Figure 1). In Phase 1, a bottom‐up approach was used so that PW themselves could direct the research agenda by identifying the topic area in which they had unmet needs. PW identified obesity as the priority topic area in which to develop a research agenda by identifying obesity‐related concerns and unmet needs (under review). Phase 2 gathered additional information on the issues that PW had concerning obesity to provide a more comprehensive picture of their obesity health concerns and unmet needs (linked article). The objective of Phase 3, reported in this article, had two stages. Part 1 was to generate additional input from multisectoral stakeholders (MS) as it is good practice in research priority setting to involve a range of stakeholders in the process.^33^ Part 2 was a ranking exercise, which only involved PW, to determine their top 10 obesity health priorities to develop an obesity research agenda. Part 2 was organized deliberately this way to address issues of power and to ensure that women's voices were not silenced to align with the principles of FPAR, which aims to transform spaces in a manner that promotes authentic power sharing and opposes gendered, racialized and class power relations.^34^

### 2.2 Survey of MS (Phase 3, Stage 1)

To align with quality research priority setting exercise practices,^33^ a survey was developed to obtain the perspectives of a wide range of MS with an interest in obesity in this population. Survey development was based on a previous survey that identified the research priorities around what makes children happy and healthy in Bradford with a range of stakeholders (unpublished survey). H. I. had assisted in the development of that survey, and it had been screened by a core group of researchers to ensure suitability for determining research priorities from a range of stakeholders. Respondents were asked to list their three most significant research priorities (an unmet needs or topic area) that should be addressed in the area of overweight and obesity in PW (File S1).

#### 2.2.1 Participants and recruitment

Participants were recruited using purposive and snowball sampling to ensure broad representation of stakeholders across different sectors including general practitioners, nurses, midwives, dieticians and nutritionists, local government workers, councillors, religious leaders and staff from health clubs and community organizations. They were approached by email and consent was obtained through a tick box included with information at the beginning of the survey.

#### 2.2.2 Analysis

NvIvo12® was used to manage data, which were analysed using inductive qualitative content analysis with a quantitative component. Content analysis followed Parveen and Showkat's^35^ approach involving four steps: (a) identify units of meaning, (b) label equivalent units with a code, (c) group similar codes into a category and (d) describe related categories with a theme. After qualitative analysis of the content, themes were quantified by counting the frequencies.^35^ To obtain an overall impression, survey responses were read and reread and discussed with all authors. H. I. checked the codes for consistency and agreement, and any differences were resolved by an iterative process.

#### 2.2.3 Statements

**Figure 2:**
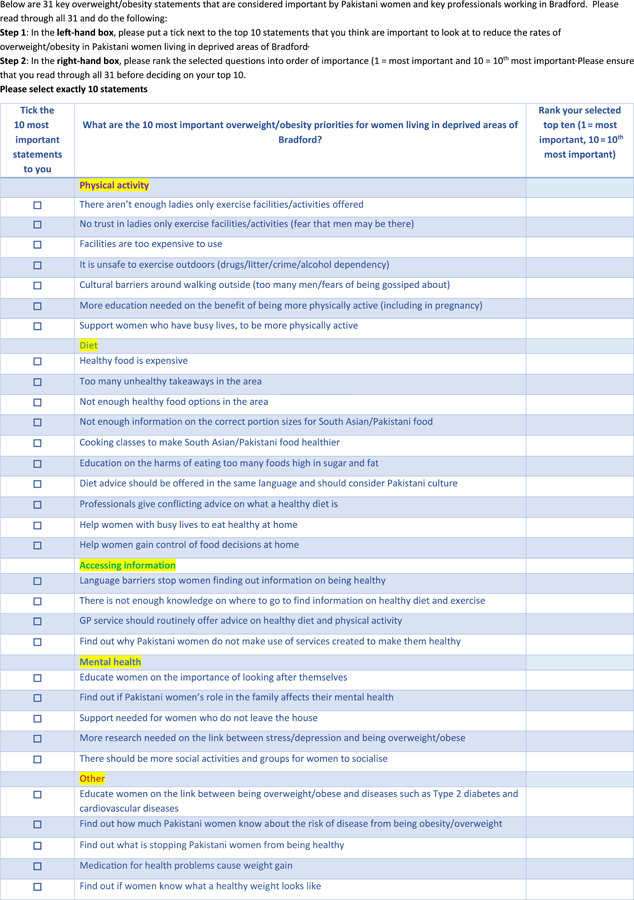
Ranking form.

A set of statements had been previously developed by PW from the earlier stages of the project. Also, the findings from the survey in the present study were translated into statements, to be ranked by PW in the final phase of the study. A total of 31 statements were categorized under five domains: diet; physical activity; accessing information; mental health; and other. A ranking form was developed containing the statements (see Figure 2).

### 2.3 Ranking exercise with PW (Phase 3, Part 2)

Only PW were invited to participate in the ranking exercise. This was to avoid difficulties that may arise conducting a joint prioritization exercise between professional stakeholders and PW, such as the stifling of women's voices due to power dynamics. Participants were also recruited as participant researchers as FPAR advocates women's involvement in data collection^36^, ^37^ to share control of the research processes with participants.^38^

#### 2.3.1 Setting and participants

Due to the lockdown resulting from the COVID‐19 outbreak, the ranking exercise could not be carried out face to face, in a group format, as planned. Instead, participants were given the option to complete the ranking exercise, individually, over voice or video call. Purposive sampling and snowball sampling were used to recruit a broad sample of participants. Five women who had taken part in Phase 2 (linked article) had expressed a desire to be involved in the ranking exercise. They were sent a participant information sheet via WhatsApp. They were asked to use their personal networks to inform others who were eligible to partake: Muslim, PW, over 18 years of age, who lived in deprived areas of Bradford. Potential participants contacted the first author to express an interest in taking part and were emailed a participant information sheet and the ranking form to view before the ranking exercise. Three non‐English‐speaking women participated in the ranking study. They were older relatives of the five participants who translated the information sheet and ranking form for their relatives.

#### 2.3.2 Data collection

Nine peer researchers were trained: the original five volunteers and a further four women who volunteered after they had participated in the ranking exercise. Training involved H. I. clarifying what each statement meant and how to guide participants through the ranking exercise. Six participants wanted to rank independently, without a researcher supporting them, and electronically return the completed form. Before their independent ranking, H. I. clarified what each statement meant. Table 1 shows how women were recruited into the study and the method by which they ranked the statements. We aimed to be flexible with ranking session timings, taking a 24/7 approach because children were being home‐schooled due to lockdown restrictions and Ramadan occurred during this phase of the study.

**Table 1:** How women were recruited into the study

Participants and recruitment method	Researcher who supported the ranking process
Existing participants from Phase 2 (*n* = 5)	H. I. (*n* = 5)
Snowball sampling by participants in Phase 2 (*n* = 14)	H. I. (*n* = 5) Participant researchers (*n* = 5) Participants individually ranked (*n* = 4)
Participants new to the study (*n* = 13)	H. I. (*n* = 7) Participant researchers (*n* = 4) Participants individually ranked (*n* = 2)

Each session started with an introduction to the ranking exercise. All 31 statements on the ranking form were read aloud to participants to ensure understanding. Participants were asked to first select the 10 statements they felt were most important in reducing the obesity rates of PW living in deprived areas of Bradford. Participants were then asked to rank their 10 selected statements from 10 to 1, with 10 being the least important and 1 being the most important.

#### 2.3.3 Analysis

A reverse scoring system was applied to each statement using the James Lind Alliance method of collating and scoring interim priorities.^27^ The statements were ranked and a score was assigned based on its position. The highest ranking (1) was assigned a score of 10. The second highest ranking (2) had a value of 9, and so forth. Position scores were added to work out their position on the list.

##### 2.3.3.1 Developing the statements into an obesity research agenda

Typically, at the final stage of research priority setting, statements are translated into researchable questions and edited for clarity,^39^, ^40^, ^41^ often by a working group.^33^ However, when translating the statements into researchable questions or priorities, misrepresentations of statements may occur, thus failing to reflect the needs of those whose very voices it intends to capture.^42^ PW, therefore, who had not been involved in the project, yet shared similar characteristics to the participants, were consulted to develop the research agenda from the prioritized statements.

#### 2.3.4 Ethical approval

Ethical approval was obtained from the University of Bradford on 12 June 2019 for Phase 3 involving the multisectoral professionals and on 9 May 2020 for the ranking exercise with PW in Phase 4 (reference E722).

## 3 RESULTS

### 3.1 Phase 3: Survey to MS

A total of 159 multisectoral professionals working in Bradford completed the survey. Respondent demographics are shown in Table 2. Respondents were largely female, from Black and minority ethnic backgrounds and worked in the healthcare sector. Under profession, the ‘other’ option was selected by academics, researchers, imams and local authority workers.

**Table 2:** Demographic and other personal information of survey respondents (*N* = 159)

Variable	*n*	%
Gender		
Female	128	80
Male	30	19
Missing	1	1
Ethnicity		
White British	61	39
White Irish	1	1
Mixed/multiple ethnic groups: White and Black Caribbean	2	1
Asian/Asian British: Indian	14	9
Asian/Asian British: Pakistani	66	42
Asian/Asian British: British Bangladeshi	3	2
Asian/Asian British: Chinese	3	2
Black British: African	3	2
Other	4	3
Missing	2	99
Sector		
NHS	86	55
Clinical	50	31
Nonclinical	36	23
Voluntary, community and social enterprise	41	26
Sports	12	8
Other	21	13
Working with Pakistani women face to face		
Yes	144	91
No	10	6
Missing	5	3

Abbreviation: NHS, National Health Service.

Content analysis of open‐ended responses identified three main themes: health behaviour barriers, education needs and mental well‐being. Many of the responses did not indicate research priorities, yet were observations made by respondents, requiring more attention. The topics covered under each theme are shown in Table 3.

**Table 3:** Topics

Healthy behaviour barriers	Education needs	Mental well‐being
*Dietary*: High calorific content of South Asian foods, overabundance of unhealthy takeaways; the high cost of healthy eating; lack of local healthy food provision; ascertain what dietary barriers persist. *Physical activity*: Lack of culturally appropriate exercise facilities; high cost of exercise facilities; lack of safety in outdoor places; women's negative attitudes towards physical activity; ascertain what physical activity barriers persist. *Language:*Lack of understanding health promotion messages and dietary advice in English; issues accessing services targeted at obesity reduction; a need to translate weight management information into written Urdu. *Interpersonal family barriers:*Influence over food decisions; busy lives and diet; busy lives and exercise.	*Diet*: Cooking classes on how to make Asian foods healthier; the harms of consuming high‐sugar and high‐fat foods typical in the South Asian diet. *Physical activity:*Information on the benefit of engaging in physical activity and safe exercise in pregnancy. *Obesity health risks*: The risk of developing diseases associated with obesity; the link between obesity and disease; ascertain whether women know about obesity health risks.	Exploration of associations between obesity and depression; exploration of the impact of stress on obesity within Pakistani women. *Mental health support:*Encouragement to attend counselling provision. *Support groups:*Setting up support groups for women to socialize. *Family dynamics:*The impact of women's role in the family on their mental health.

#### 3.1.1 Theme 1: Healthy behaviour barriers

Healthy behaviour barriers were most frequently mentioned by survey respondents (*n* = 125) as a concern for PW in managing their weight. These included (i) dietary barriers, (ii) physical activity barriers, (iii) language barriers and (iv) interpersonal family barriers. Respondents reported the need to ascertain from women directly their barriers in engaging in healthy dietary behaviours:

*Find out from women why they struggle to eat healthily*.

Nearly a third of respondents (*n* = 47), however, reported a range of dietary barriers to healthy eating. These included the high calorific content of South Asian foods, making it difficult for women to select a healthy diet:

*The cultural food they eat is typically high in calories*.

Many participants outlined barriers in obtaining healthy food in PW's local area. An overabundance of unhealthy takeaways locally, the high cost of healthy eating and the lack of local healthy food provision were also mentioned as areas requiring more attention for PW to be able to manage their weight:

*Healthy food availability in poorer areas is lacking*.

Similar to the dietary barriers reported above, respondents were also unsure of the barriers that prevented women from being physically active:

*Find out from them the main obstacles in engaging in physical activity*.

A quarter of respondents (*n* = 41) highlighted a range of barriers faced by PW when it came to undertaking regular physical activity. These included the lack of culturally appropriate and expensive facilities promoting physical activity engagement:

*issues around purdah (modesty) and free mixing in gyms and other exercise centres*.

Respondents highlighted environmental barriers in local areas that acted as a deterrent for women from engaging in exercise to control their weight:

*Safety of outdoor places to walk in their areas*.

Individual factors were also perceived to be an obstacle in weight management in PW.

*Their attitudes towards physical activity is why they're so unhealthy*.

Respondents (*n* = 17) observed that language barriers hindered their ability to obtain information on weight management and the need to translate weight management information into a language understood by women.

*The information needs to be written in Urdu so they can understand it*.

Interpersonal family barriers were reported by 20 respondents and included the influence of family members on making food decisions in the home:

*Asian men (husbands) want foods filled with oil/heavy to satiate their hunger so request it often*.

Also reported were concerns about the busy lives that PW lead and how it poses as a barrier to living a healthy lifestyle.

*Competing priorities (busy Asian lifestyle) so finding time to work out and eat healthy isn't easy*.

#### 3.1.2 Theme 2: Education needs

Fifty‐one respondents indicated an unmet need for obesity‐related education around diet, physical activity and obesity health risks. In particular, respondents mentioned that PW were unaware of what foods constituted a healthy diet and their quantities:

*healthy foods/unhealthy food e.g., many Pakistani women will happily consume lots of nuts or fruit juices thinking this is healthy and low in fat/sugar when in fact it may cause weight gain*.

Participants disclosed the need for cooking classes:

*Teach them how to cook Asian food so it's healthier but tastes good*

and education on the harm of eating unhealthy cultural food, teaching about low‐calorie food options and what foods to incorporate into their diet:

*Education on harm of consuming foods high in sugar and fat like is typical in their diet. They don't seem to have this awareness*.

Respondents also highlighted that women required information on the benefit of engaging in physical activity and, more specifically, education that exercise is safe in pregnancy.

*They need to be educated/informed that there is real benefit to exercising for their mental and physical state. If they knew, it could help them lose weight and feel better about themselves*.

*They think it's dangerous to exercise while pregnant. There is an education need there*.

Additionally, respondents mentioned that women need education to understand obesity health risks:

*Education on what causes diabetes and other diseases that lots of Asians have in Bradford*.

#### 3.1.3 Theme 3: Mental well‐being

Mental health was identified by respondents as a topic area requiring more attention. This included whether poor mental health impacted obesity in PW:

*Research needs to examine if there's a link between obesity and depression in this group*.

Respondents noted that some women are socially isolated, tending to stay indoors and thus not engaging in behaviours conductive to a healthy weight:

*I've noticed through my work that many of them stay home most of the time so they're just gaining weight*.

Some respondents stated that many PW do not leave the house due to cultural constraints, with family members dictating whether women could leave the house:

*Some women aren't allowed to go out or its disliked by husband, family and inlaws*.

Respondents also noted a lack of support groups for women to socialize:

*Availability of support groups/setting up of support groups. So they don't feel isolated and know lots of other women in the same situation*.

It was stated that women needed to be encouraged to access mental well‐being support:

*More encouragement for them to go for counselling as they are very reluctant*.

Women's role in the family in relation to mental well‐being was also identified as an area that required more research.

*Ask women if their cultural role in the family impacts their mental health*.

### 3.2 Phase 4: Ranking exercise results

Thirty‐two women completed the ranking forms. Participants lived in the most deprived wards of Bradford, which fall within the 10% most deprived wards in England according to the UK Indices of Deprivation data.^13^ They were aged between 18 and 69 years and were mostly English‐speaking. Three were proficient in spoken Urdu and could also read Urdu. See Table 4 for participants' demographics.

**Table 4:** Demographics of ranking exercise participants

Participant demographics	No. of participants (*n* = 32)
Participant age	
18–28	8
29–39	12
39–49	7
49–59	2
59–69	3
English‐speaking	29
Non‐English‐speaking	3

Table 5 below shows a summary of the 31 obesity statements ranked in order of priority. The ranking score was calculated in the first right column next to each statement. The second column shows whether the statement was identified by PW in Phase 1/2 or both, by MS in the present study or both women and stakeholders. The third column shows the number of participants that selected the statement. Some statements were selected by more participants than those that were ranked higher but were assigned a lower score, thus ranking lower overall. There were large discrepancies between statements that scored high and those that scored low. Four of the top 10 statements belonged in the domain of *mental health*, two statements in the domain of *physical activity*, two in the domain of *diet*, one was within the domain of *accessing information* and one statement in the list did not belong to any domain. The statements are ordered in terms of priority based on their ranking score.

**Table 5:** Summary of ranked statements

Rank order	Statement	Ranking score	Identified by whom?^a^	No. of times selected
1	Find out if Pakistani women's role in the family affects their mental health	115	MS	16
2	Support needed for women who do not leave the house	109	PW2	19
3	Educate women on the importance of looking after themselves	96	PW2	15
4	More research needed on the link between stress/depression and being overweight/obese	93	MS	13
5	Education on the harms of eating too many foods high in sugar and fat	92	PW2/MS	11
6	Educate women on the link between being overweight/obese and diseases such as Type 2 diabetes and cardiovascular diseases	91	MS	13
7	More education needed on the benefit of being more physically active (including in pregnancy)	85	MS	15
8	GP should routinely offer advice on healthy diet and physical activity	72	PW1	11
9	Diet advice should be offered in the same language and should consider Pakistani culture	71	PW2/MS	13
10	No trust in ladies‐only exercise facilities/activities (fear that men may be there)	69	PW2	11
11	Healthy food is expensive	68	PW1/MS	10
12	Too many unhealthy takeaways in the area	68	PW2/MS	15
13	Find out if women know what a healthy weight looks like	65	PW2	12
14	Medication for health problems causes weight gain	64	PW2	15
15	Support women who have busy lives to be more physically active	57	PW2/MS	11
16	Find out why Pakistani women do not make use of services created to make them healthy	55	MS	11
17	Cultural barriers around walking outside (too many men/fears of being gossiped about)	53	PW1&2/MS	12
18	Not enough information on the correct portion sizes for South Asian/Pakistani food	51	PW2	9
19	Find out what is stopping Pakistani women from being healthy	49	MS	8
20	Help women with busy lives to eat healthy at home	45	PW2/MS	8
21	Find out how much Pakistani women know about the risk of disease from being overweight/obese	43	MS	6
22	Not enough healthy food options in the area	40	PW1&2/MS	8
23	Help women gain control of food decisions at home	38	PW2/MS	8
24	Language barriers stop women finding out information on being healthy	36	PW1&2/MS	5
25	There should be more social activities and groups for women to socialize	32	MS	6
26	Cooking classes to make South Asian/Pakistani food healthier	24	PW2/MS	7
27	Facilities are too expensive to use	21	MS	6
28	There are not enough ladies‐only exercise facilities/activities offered	20	PW1/MS	4
29	It is unsafe to exercise outdoors (drugs/litter/crime/alcohol dependency)	19	PW1&2/MS	5
30	There is not enough knowledge of where to go to find information on healthy diet and exercise	10	PW1	4
31	Professionals give conflicting advice on what a healthy diet is	9	PW2	3

*Note*: PW1: Pakistani women (Phase 1, in review). PW2: Pakistani women (Phase 2, linked article). MS: multisectoral stakeholders (Phase 3, current study).

^a^ Statements identified by Pakistani women were identified in previous phases of the research.

## 4 DISCUSSION

### 4.1 Summary of the findings

A range of topic areas and unmet needs were identified by stakeholders. Many of the findings, however, were not research priorities, yet were observations as to why PW struggled to manage their weight and what they felt was needed by this population to overcome obesity. This may have been because some survey respondents may not have known what was meant by research priorities as was reported in other research priority setting exercises involving stakeholders from a nonclinical background.^31^, ^43^, ^44^ Findings concerned healthy behaviour barriers such as dietary, physical activity, language and interpersonal barriers, the need for obesity‐related dietary education, physical activity and obesity health risks and issues concerning mental well‐being. The main findings from the survey were translated into statements, to be ranked by PW. Greatest priority was given to statements concerning the need for education around mental health, physical activity and diet.

### 4.2 Similarities and differences between PW and MS

Similarities and differences could be found between topics identified by stakeholders and those by PW. Notably, the four statements identified by stakeholders around the reasons why women did not engage in healthy behaviours were not ranked highly by women. This could be because women may already feel that these barriers are well known. Surprisingly, the statements relating to the high cost of food and facilities being too expensive to use were not in the prioritized list, despite the women living in deprived areas. This could either be because PW did not perceive finances to be a barrier in engaging in health‐promoting activities, or because they felt that other concerns were more pressing. These differences highlight the importance of involving underrepresented communities in research priority setting whose views are traditionally not considered.

PW assigned the highest rankings to four of five available statements surrounding mental health. This was surprising, given that two of the mental health‐related statements were not identified by PW in the earlier studies, yet were instead identified by stakeholders in the present study. This demonstrates the importance of involving a variety of different stakeholders and sources in health research prioritization to ensure that a range of perspectives are incorporated into the process, advocated by the research priority setting literature.^33^, ^45^ PW in earlier studies of this project may not have considered mental health to be significant in relation to obesity, but recognized its importance when they saw it on the ranking form. Many of the stakeholders were female and from Black and minority ethnic backgrounds, reflecting the diverse ethnic profile of Bradford. This may have influenced the findings, as these respondents may have had more insight into issues encountered by PW living in deprived areas of Bradford.

### 4.3 Social determinants of health

The findings from this study demonstrate that obesity and overweight extend beyond the responsibility of the individual and the multifaceted layers of influence that impact an individual such as socioeconomic status, culture, religion, family influence and community networks need to be considered. Health inequalities are not always considered in obesity research, and to ensure successful intervention design, understanding the socioeconomic situations of those with obesity is essential,^46^ as is the need to address structural barriers to bring about behaviour change. Dahlgren and Whitehead's^46^ rainbow model provides a useful framework for exploring the interaction between the determinants and their influence on obesity in PW living in deprived areas of Bradford. Our findings can be contextualized within the layers of influence reported by the model, especially *individual lifestyle factors, social and community networks* and *living and working conditions*.

In the model, individual lifestyle factors relate to behavioural risk factors, which are potentially modifiable, such as low uptake of physical activity in PW and poor dietary habits, which are known contributors to obesity.^47^ It is argued that there is an element of choice in becoming obese.^48^ Our findings, however, suggest that these women do not have much choice in managing their weight due to the structural barriers that they encounter such as healthy food and exercise facilities being expensive, unsafe neighbourhoods for physical activity and a lack of culturally appropriate dietary advice.

The second layer is the collective social context, that is, mutual support from family, friends, neighbours and the local community, which are external to the individual, yet influence the level of health experienced in that population.^46^ International evidence suggests that family social capital, that is, positive close relationships with household members, can have health‐promoting aspects.^49^ Our findings suggest that PW interact with their family, peers and community, which, in turn, influences their health behaviours. Prioritized interpersonal barriers included the need to ascertain whether PW's role in the family affects their mental health, and support needed for women who do not leave the house. However, it has been previously reported that in deprived areas of Bradford, PW are more likely to report low family social capital compared to White British women.^50^

The third layer of the rainbow is an individual's ability to maintain their health and is influenced by living and working conditions, food supply and access to essential goods and services.^46^ A range of structural issues were identified by survey respondents that made it difficult for women to manage their weight, and were ranked as important by PW. These included accessing weight management services for non‐English‐speaking and socially isolated women as well as the prioritized statements on education.

#### 4.3.1 Prioritized statements

A research agenda was developed from the prioritized top 10 statements, with public and patient involvement (PW from deprived areas of Bradford). The research priorities are italicized below and are shown as an obesity research agenda in Table 6 below.

**Table 6:** An obesity research agenda for Pakistani women living in deprived inner‐city areas of Bradford

	Obesity research agenda
1	Explore the relationship between Pakistani women's role in the family and their mental health, and the impact that this has on obesity.
2	Identify and test culturally and economically appropriate ways in which socially isolated Pakistani women can be supported to manage their weight.
	Determine the best ways to deliver mental health support to socially isolated Pakistani women living in deprived inner‐city areas of Bradford.
3	Explore the best ways of educating Pakistani women living in deprived inner‐city areas of Bradford on the importance of looking after themselves.
	What are the different ways in which health promotion education can be delivered that is impactful to Pakistani women living in deprived inner‐city areas of Bradford?.
4	identify ways in which information on depression and obesity can be best communicated with Pakistani women.
5	Consider education and literacy levels when designing health education programmes for Pakistani women living in deprived inner‐city areas of Bradford, so that those receiving the education find them appropriate to their language skills.
6	Explore the best ways of delivering culturally and religiously appropriate educational interventions to Pakistani women living in deprived inner‐city areas of Bradford.
7	Determine whether health promotion messages are understood by Pakistani women living in deprived inner‐city areas of Bradford.

##### Mental health

The top four ranked prioritized statements all related to mental health. A study aimed at identifying research priorities for public health research also found that mental health was ranked the highest by participants, particularly ethnic minority groups.^51^ Our findings were unsurprising as PW suffer exceptionally poorer mental health.^52^ The relationship between obesity and mental health issues is more pertinent in women than men,^53^ with social isolation a feature of the experiences of depressed PW.^54^ There is a correlation between high social exclusion and adverse health outcomes, particularly relating to mental health.^55^ Thus, to address the priority *support needed for women who do not leave the house*, research is needed to *identify and test culturally and economically appropriate ways in which socially isolated PW can be supported to manage their weight*. Mental health services are also underutilized by this population^52^ as well as low‐income groups more generally.^56^ Social isolation prevents women from accessing mental health support outside the house. To address this, research is needed to *determine the best ways to deliver mental health support to socially isolated PW living in deprived inner‐city areas of Bradford*.

Another priority was for the need for more research to ascertain the link between stress/depression and being overweight/obese. Existing evidence highlights the association between depression and obesity, especially in women.^57^ Chronic stress is associated with obesity and its resulting illnesses as it is known to alter food intake patterns, and dietary preferences.^58^ Despite a plethora of evidence linking stress/depression and being overweight/obese, the statement was still prioritized by PW. This suggests that PW are unaware of information surrounding depression and obesity. A research priority, then, is to *identify ways in which information on depression and obesity can be best communicated with PW*.

Research is needed examining the highest‐ranking priority: Whether PW's role in the family affects their mental health in relation to overweight and obesity. The literature has found a positive association between a range of family stressors and overweight and obesity in children,^59^ and associations between cultural gender roles in the family and obesity and overweight in Moroccan women.^60^ No research was found related to PW; thus, research is needed to *explore the relationship between PW's role in the family and their mental health, and the impact that this has on obesity*.

##### Education

Four of the top 10 ranked priorities concerned the need for education around a healthy diet, increasing physical activity levels, the disease risk from obesity and educating women on the importance of looking after themselves. It is fundamental to ensure that educational interventions have practical relevance and are sensitive to the contextual and cultural characteristics of target populations^61^ as health promotion messages are more likely to be effective if they consider the lived experiences, values and beliefs of individuals.^62^ Research should *explore the best ways of delivering culturally and religiously appropriate educational interventions to PW living in deprived inner‐city areas of Bradford*. Priority was given to dietary advice to be offered in the same language, including translating written materials. In our earlier study (Phase 2) (linked article), it was identified that some UK PW cannot read Urdu. Therefore, research must *explore the best way to deliver weight management advice to non‐English‐speaking women living in deprived inner‐city areas of Bradford*.

### 4.4 Strengths and limitations

A strength of this priority setting exercise is the incorporation of views from professional stakeholders across different sectors, including religious leaders and fitness professionals, who, to our knowledge, have previously been uninvolved in research prioritization. Another strength is that PW were solely involved in the ranking exercise rather than a joint prioritization exercise with other stakeholders, which could have stifled their voices. Instead, they were able to rank the statements without any pressure to conform. Also, PW translated the obesity health concerns and obesity unmet needs into research priorities rather than members of the research team. This reduced the risk of statements being misinterpreted. Lastly, by recruiting non‐English‐speaking women, we aimed to ensure that this study would be applicable to the wider British Pakistani population.

Limitations include the top 10 list, which could have been different had the priority setting exercise been set up differently such as disagreements bring resolved through facilitated debate and lists prioritized based on consensus.^27^ However, the COVID‐19 pandemic lockdown made it impossible to hold a face‐to‐face consensus exercise. Another limitation is that scores between some of the ranked statements were marginal, which meant that a statement could have switched positions in the list had one participant ranked differently. This needs to be considered in future research priority setting as there are implications involved in relying on prioritized lists to develop projects or interventions. Also, the researcher was not present during all 32 ranking sessions. As a result, there was uncertainty around the manner in which the participant researchers may have influenced the ranking, despite being trained on how to facilitate the exercise. Lastly, depth was lacking in some of the survey responses, making it difficult to draw conclusions from what was stated. Therefore, future research should be conducted with PW to further understand how these priorities are interpreted.

## 5 CONCLUSION

This study has reported on the unmet obesity needs and topic areas requiring more research in relation to PW living in deprived areas of Bradford, according to the women and MS. Findings showed that education needs, healthy behaviour barriers, mental well‐being and the need to obtain women's perspectives on why they do not engage in weight management behaviours were key areas that required research focus. PW prioritized statements concerning education needs, and mental health. The prioritized statements were developed into an obesity agenda by PW. Actively engaging UK PW in setting research priorities provided a unique opportunity to understand the key areas they think are important for future research. The culminating research agenda can be used by researchers to advance the field of obesity research in Pakistani communities, thus producing research outputs that are relevant to this population.

## AUTHOR CONTRIBUTIONS

Halima Iqbal conceived the original idea and designed the study. Halima Iqbal managed the project and collected data. All the authors have contributed to the interpretation of data. Halima Iqbal drafted the manuscript, and all authors were involved in revising the manuscript and have given final approval of the version to be published.

## CONFLICTS OF INTEREST

The authors declare no conflicts of interest.

## Supporting information

Supplementary information.

## Data Availability

The data that support the findings of this study are available from the corresponding author upon reasonable request.
