# Supplementary material for: Developing an obesity research agenda with British Pakistani women living in deprived areas with involvement from multisectoral stakeholders: Research priority setting with a seldom heard group
Source: Health Expect. 2022 Apr 28;25(4):1619–32. doi: 10.1111/hex.13504 (PMC9327852; doi:10.1111/hex.13504)
Supplement: Supplementary file 1 — Supplementary information. [file HEX-25--s001.docx]

# Supplementary file 1: Survey

**Page 1: Participant Information Form**

**Project Aim**

The purpose of this quick survey is for a wide range of professional stakeholders to identify topic areas or questions around overweight and obesity in Pakistani women living in deprived areas of Bradford that they feel requires more research (research priorities).

**Where this survey fits in**

This research is being undertaken as part of a PhD. The aim is to use a systems wide approach to collect a broad range of perspectives from Pakistani women and professional stakeholders, to identify areas that require more research in overweight and obesity for Pakistani women.

I have already conducted interviews and focus groups with 42 Pakistani women living in deprived areas of Bradford to identify and explore the areas they feel are important for research to address when it comes to overweight and obesity.

The next stage of the research is *this* survey which aims to ascertain what a range of professional stakeholders perceive to be important areas that need more research around overweight and obesity in Pakistani women living in deprived areas of Bradford.

The final stage of the research will be a survey to rank the collective research priorities identified by both Pakistani women and professional stakeholders.

**Why have I been asked to take part?**

This survey is targeted at a wide range of professionals working in Bradford that have an interest in overweight and obesity in Pakistani women living in deprived areas of Bradford.

You will be asked for consent before starting the online survey. You will have the option at the end of providing an email address if you wish to be considered for involvement in the ranking survey (the final stage of the study)

**Confidentiality**

Whilst some demographic details will be collected in order to understand your working background, no personal details will be recorded, and it will not be possible to identify you in the thesis or in dissemination of findings.

**Data Storage**

The delivery system used for the survey is JISC online surveys, the adopted delivery system at the University of Bradford. The stored data is password protected and it will only be accessible by the researcher.

**Ethics Committee Clearance**

The project has been approved by the Ethics Committee at the University of Bradford (2019).

**Queries and Concerns**

If there are any queries or concerns about the survey, please contact the researcher.

**Researcher**Name: Halima Iqbal

Faculty of Health Studies, University of Bradford, UK

Email: [hiqbal23@bradford.ac.uk](mailto:hiqbal23@bradford.ac.uk)

**Consent**

In line with ethical approval, consent is needed before completing the survey. By selecting yes, you are confirming that you have read the guidance and consent to your answers being used as part of a PhD thesis and related publications. By selecting yes, you are also confirming that you work in a setting within Bradford and have an interest in overweight and obesity in Pakistani women.

- - - 1. I consent to undertake the survey and confirm I meet the inclusion criteria


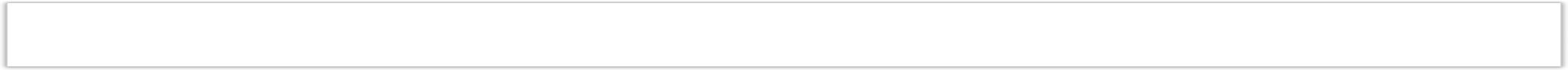

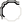


Yes

**Page 2: Background**

The purpose of this section is to ascertain your background. The data produced will give an insight into the diversity of survey respondents and the setting in which they work.

- - - 1. What is your job title?


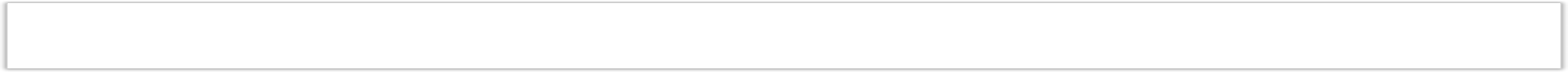


- - - 1. Are you responding to this survey as a (please tick all that apply)


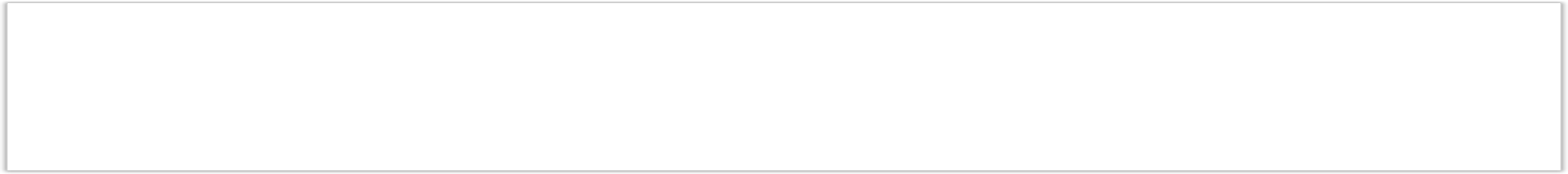


Researcher

Academic

Professional that works with Pakistani women and their families

Other

*3.a.* Which of these categories below best describes your profession?

 More info


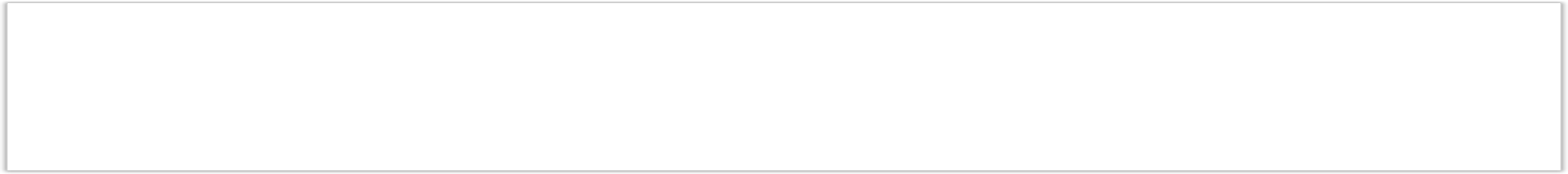


Health care professional

Care/community/voluntary sector professional

Sports professional

Other

*3.a.i.* If you selected Other, please specify:


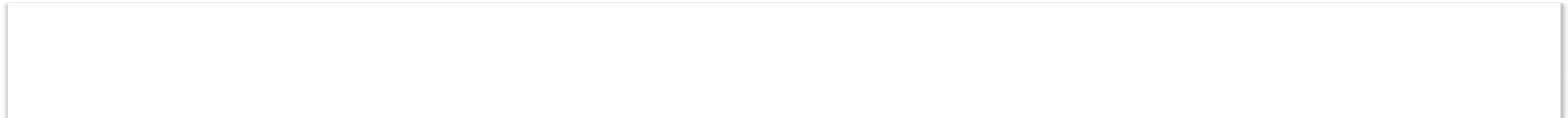


- - - 1. Do you work with Pakistani women face to face?


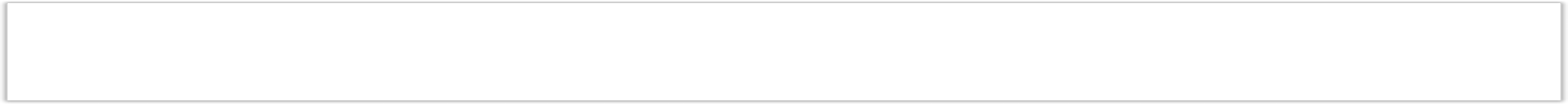


Yes

No

- - - 1. What is your age?


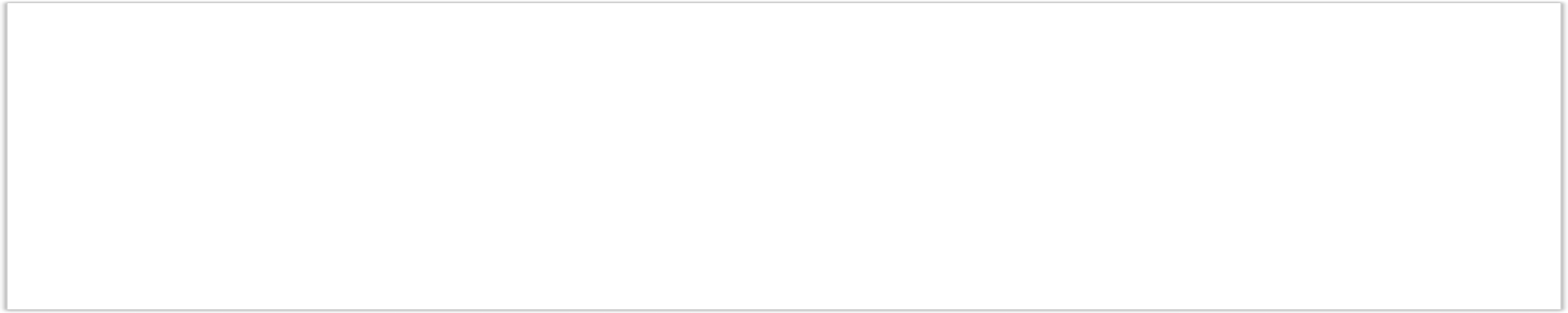


11-20

21-30

31-40

41-50

51-60

61-70

71-80

80 and above

1. Are you:


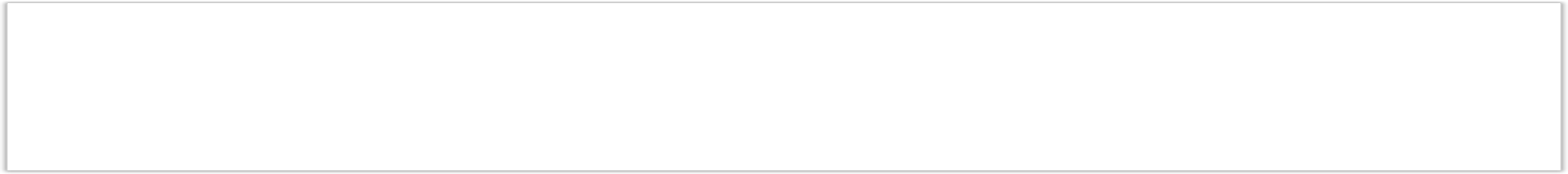


Male

Female

I prefer to use my own term

I prefer not to say

1. What is your ethnic group?


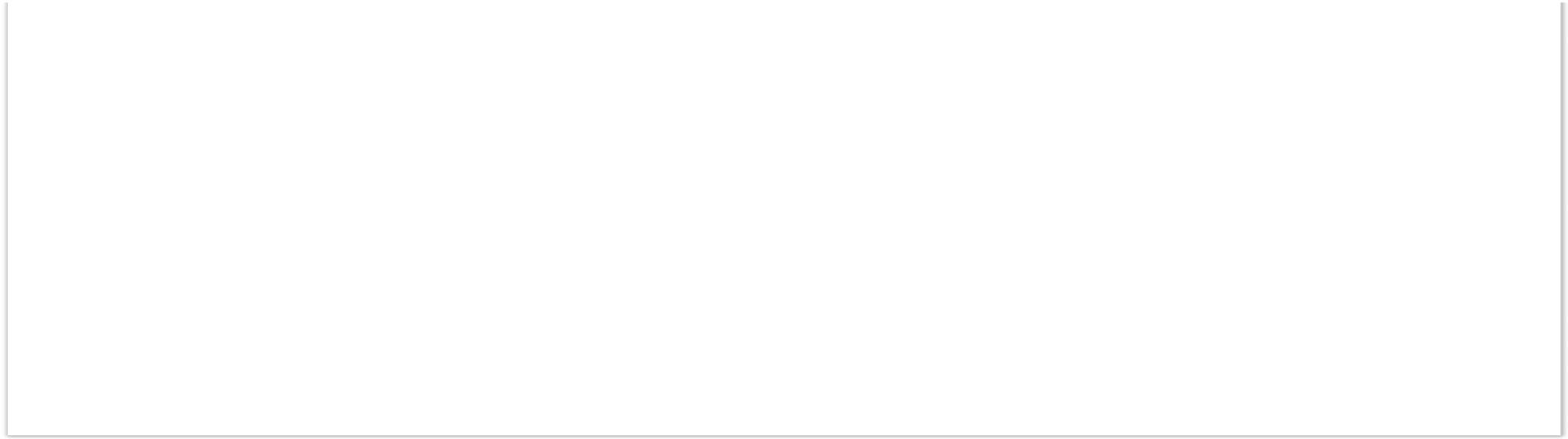

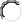

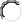

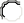

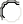

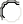

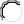

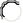

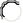

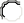

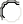

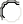

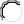

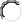

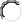

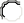

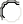

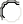

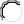


White:

White: Irish

English/Welsh/Scottish/Northern

Irish/British

White: Gypsy or Irish

Traveller

Mixed/Multiple ethnic groups: White and Black Caribbean

Asian/Asian British: Pakistani

Black British: African

Eastern European: White; Czech

White; Gypsy/Roma

Mixed/Multiple ethnic groups: White and Asian

Asian/Asian British: Bangladeshi

Black British: Caribbean

White; Romania

Asian/Asian British:

Indian

Asian/Asian British: Chinese

Eastern European: White; Polish

White: Slovakia

Other ethnic group:

Arab/Middle Eastern

Other

*7a*. If you selected Other, please specify:


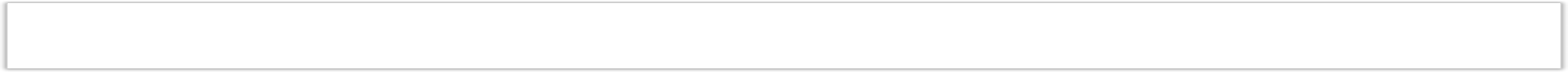


**Page 3: Identifying obesity research needs**

1. The purpose of this section is to generate obesity research priorities for Pakistani women living in deprived areas of Bradford.

Please answer the following questions: What do you feel are the top **three** most significant research priorities (an unmet need or topic area) that should be addressed in the area of overweight and obesity in Pakistani women?


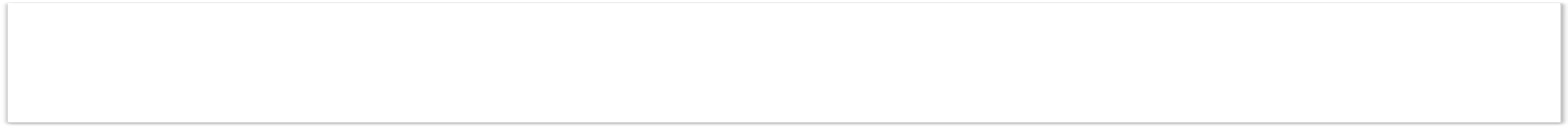


This could be in relation to:

- diet
- physical activity
- environment

1. Are there any other areas or questions you feel future research should examine in terms of overweight and obesity in Pakistani women living in deprived areas of Bradford?


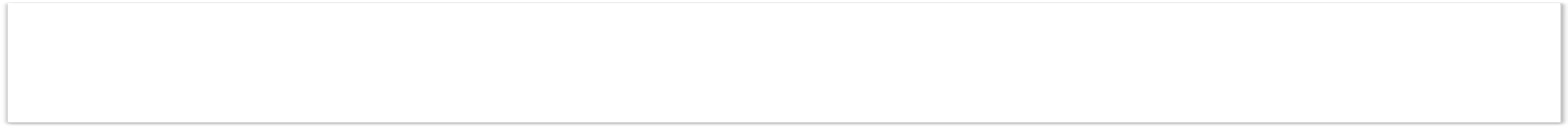


Thank you for taking part in the survey.
